# Crystal structure of Gib2, a signal-transducing protein scaffold associated with ribosomes in *Cryptococcus neoformans*

**DOI:** 10.1038/srep08688

**Published:** 2015-03-03

**Authors:** Rya Ero, Valya Tenusheva Dimitrova, Yun Chen, Wenting Bu, Shu Feng, Tongbao Liu, Ping Wang, Chaoyang Xue, Suet Mien Tan, Yong-Gui Gao

**Affiliations:** 1 School of Biological Sciences, Nanyang Technological University, Singapore; 2 Public Health Research Institute, Department of Microbiology and Molecular Genetics, Rutgers University, Newark, NJ, USA; 3 The Research Institute for Children, Children's Hospital, New Orleans, LA, USA; 4 Department of Pediatrics, Louisiana State University Health Sciences Center, New Orleans, LA, USA; 5 Department of Microbiology, Immunology, and Parasitology, Louisiana State University Health Sciences Center, New Orleans, LA, USA; 6 Institute of Molecular and Cell Biology, A*STAR, Singapore

## Abstract

The atypical Gβ-like/RACK1 Gib2 protein promotes cAMP signalling that plays a central role in regulating the virulence of *Cryptococcus neoformans*. Gib2 contains a seven-bladed β transducin structure and is emerging as a scaffold protein interconnecting signalling pathways through interactions with various protein partners. Here, we present the crystal structure of Gib2 at a 2.2-Å resolution. The structure allows us to analyse the association between Gib2 and the ribosome, as well as to identify the Gib2 amino acid residues involved in ribosome binding. Our studies not only suggest that Gib2 has a role in protein translation but also present Gib2 as a physical link at the crossroads of various regulatory pathways important for the growth and virulence of *C. neoformans*.

C*ryptococcus neoformans*, an encapsulated yeast-like basidiomycetous fungus, is the primary culprit behind fungal meningoencephalitis in immune-compromised individuals^1^. *C. neoformans* afflicts approximately one million people worldwide, primarily in developing countries, and accounts for over 600,000 fatalities annually^2^,^3^. The relevance and genetic tractability has enabled *C. neoformans* to emerge as a model organism to study the molecular mechanisms of fungal pathogenesis.

Factors important for the virulence of *C. neoformans*, such as the antioxidant melanin pigment and anti-phagocytic capsule, are regulated by the conserved cAMP-dependent signalling pathway mediated by the GTP-binding (G) protein α subunit Gpa1^4^,^5^,^6^,^7^,^8^. Gpb1, the only known classical G protein β subunit in *C. neoformans*, regulates pheromone-responsive mating and haploid differentiation through association with the G protein α subunits Gpa2 or Gpa3 and γ subunits Gpg1 or Gpg2, but has not been implicated in Gpa1-cAMP signalling^9^,^10^,^11^,^12^. Although a classical G protein β subunit was not found for Gpa1 in *C. neoformans*, Palmer and co-workers reported that a Gpa1-interacting protein, Gib2, could function as an atypical G protein β subunit^13^. Gib2 binds directly to Gpa1 and likely facilitates its oscillation between the active and inactive states, thereby affecting cAMP signalling^13^. Gib2 was also shown to promote cAMP levels in cells lacking Gpa1 presumably by relieving the inhibitory effect of Ras1 protein on adenylyl cyclase Cac1 protein in the absence of Gpa1^13^,^14^. In addition to Gpa1, Gib2 physically interacts with Gpg1 and Gpg2, as well as with a downstream target of Gpa1-cAMP signalling, Smg1 protein, and several other proteins^13^,^14^.

Similar to Gpb1, Gib2 contains a seven Trp-Asp (WD) repeat motif^13^. The WD repeat family of proteins comprise polypeptide stretches of 40–60 residues that each fold into a four-stranded antiparallel β-sheet. Hence, Gib2 was likewise predicted to fold into a seven-bladed β-propeller, similar to that seen in the crystal structure of β transducin^15^,^16^. Indeed, modelling revealed that the Gib2 and Gpb1 structures were similar^13^. However, based on the amino acid sequence analysis, Gib2 is more closely related to RACK1 (receptor for activated kinase C) protein orthologues than to G protein β subunits^13^,^14^. For example, Gib2 shares 70% amino acid sequence identity with mammalian RACK1 but only 25% with *C. neoformans* Gpb1^13^. The high sequence similarity shared between *C. neoformans* Gib2 and the extensively studied human RACK1, as well as Asc1 protein (the RACK1 orthologue in *Saccharomyces cerevisiae*) (Fig. 1) suggests that Gib2 could have functions similar to those of the aforementioned proteins.

Human RACK1 orthologues are scaffold proteins that integrate numerous cellular processes (e.g., development, neuropathology, and cellular stress) through interacting with as many as 80 estimated protein partners, among which are kinases (e.g., PKC, Src, and FAK), phosphatases, membrane receptors (e.g., integrin β subunits), and G proteins^17^,^18^,^19^,^20^,^21^. RACK1 presumably recruits these proteins to their appropriate subcellular sites, thereby integrating various intracellular signalling pathways^22^. The deletion of RACK1 orthologues is lethal in higher eukaryotes, whereas the consequence of Asc1 deletion is less severe in *S. cerevisiae*^23^.

As mentioned above, Gib2 directly interacts with the G proteins Gpa1, Gpg1, and Gpg2^13^,^14^. Gib2 also physically interacts with the protein kinase C homologue Pkc1^13^. Using GST affinity purification combined with mass spectrometry, Wang and co-workers identified approximately 50 proteins that interact with Gib2, including proteins involved in signalling, intracellular trafficking, stress responses, and metabolism^14^. Interestingly, a significant proportion of the identified proteins are involved in protein translation and ribosome composition^14^. The same finding was also seen in RACK1 and Asc1 interactomes^14^,^24^,^25^. In fact, both RACK1 and Asc1 have been shown to be the ribosomal core proteins associated with the 40S ribosomal subunit^25^,^26^,^27^,^28^.

Hence, Gib2, a scaffold protein interconnecting various cellular processes through binding a myriad of proteins, could also bind with the ribosome and function in ribosomal biogenesis and protein translation. By recruiting various proteins to ribosomes, Gib2 may act as a link between protein translation and other cellular processes. To better illustrate such functions, we here present the crystal structure of Gib2 and show its interaction with ribosomes of *C. neoformans*. We also present predictions of Gib2 residues involved in the association with ribosomes and discuss the role that Gib2-ribosome binding may play in the virulence of *C. neoformans*.

## Results

### Effect of *GIB2* disruption on *C. neoformans* growth

The *C. neoformans* species includes two highly relevant but distinct varieties, var. *grubii* and var. *neoformans*. Gib2 was originally reported to have an essential function in *C. neoformans* var. *neoformans* (serotype D) because the knockdown of *GIB2* by antisense suppression resulted in a severe growth defect, and no *GIB2* deletion strains linked to the auxotrophic marker *Ura5* could be recovered^13^. However, the *GIB2* deletion strains could be readily recovered if dominant selective marker genes were used^14^. The *GIB2* deletion strains displayed no reduction in the cAMP levels or apparent defects in melanin and capsule formation, suggesting that they are not directly linked to virulence^14^. However, based on spotting a serially diluted cell culture on medium plates, the growth of the *GIB2* deletion strain was reduced at 37°C but not at 30°C or 23°C^14^. In addition, mice infected with the *GIB2* deletion strain survived nearly twice as long as those infected with the wild-type strain^14^. Apparently, although not essential, Gib2 is important for growth at the mammalian body temperature and is required for full virulence.

To accurately assess the effect of *GIB2* disruption on the viability of *C. neoformans*, growth curves of the *GIB2* deletion strain and its parental strain H99 were determined at 30°C and 37°C in rich YPD and nutrient-limiting YNB media. Although the two strains exhibited similar growth profiles at 30°C, the *GIB2* deletion strain showed approximately a two-fold reduction in growth in YPD media at 37°C (Fig. 2, left panel). This finding is in agreement with that of previous studies by Wang and co-workers^14^. The effect of *GIB2* deletion on *C. neoformans* growth is even more pronounced in YNB medium and can be observed even at 30°C (Fig. 2, right panel). It was reported previously that a higher level of Gib2 expression could be found when the cells were switched to YNB medium^13^. Thus, Gib2 is responsive to nutrient deprivation conditions.

### Ribosome binding of Gib2

Basic cellular functions, such as ribosomal biogenesis and protein translation, underlie the growth and differentiation of eukaryotic cells. Mammalian RACK1 and *S. cerevisiae* Asc1 proteins, to which Gib2 shares high homology, are core ribosomal proteins that regulate growth in response to stresses, such as elevated temperature^24^,^25^,^26^,^27^. To test whether *C. neoformans* Gib2 can form a complex with ribosomes as a basis of the thermal response, we assessed the binding of a recombinant (His-tagged) Gib2 to 80S ribosomes purified from wild-type H99 and the *GIB2* deletion strains. For comparison, the binding of human RACK1 to *C. neoformans* ribosomes was also tested. Following incubation with either Gib2 or RACK1, ribosomes were precipitated through a sucrose cushion by centrifugation, and the associated proteins were separated using SDS-PAGE. As a control, Gib2 and RACK1 proteins were loaded onto sucrose cushion in the absence of ribosomes. For reference, purified Gib2 and RACK1 proteins were directly loaded onto SDS-gel as well. The recombinant Gib2 and RACK1 were visualised by Western blotting using the anti His-tag and anti-RACK1 antibodies, respectively.

Our results revealed that the recombinant Gib2 binds to ribosomes from both wild- type (WT) and the *GIB2* deletion (*Δgib2*) strain (Fig. 3A). However, binding to the *GIB2* deletion strain ribosomes is more efficient, indicating that endogenous Gib2 protein co-purifies with ribosomes from the wild-type strain. Our data suggest that, similar to RACK1 and Asc1 proteins discussed above, Gib2 is a core component co-purifying with the ribosomes. When the endogenous Gib2 is not present in *C. neoformans* cells, the recombinant Gib2 can bind to ribosomes *in vitro*. That recombinant Gib2 can bind to ribosomes isolated from the wild-type strain, albeit less efficiently, indicates that a proportion of native Gib2 is exchanged in the binding assay, although it is possible that isolated wild-type ribosomes are not “saturated” with the native Gib2. The human RACK1 was able to bind to *C. neoformans* ribosomes (Fig. 3B), further highlighting the conservation between Gib2 and RACK1.

### Crystal structure determination

**Figure 1:**
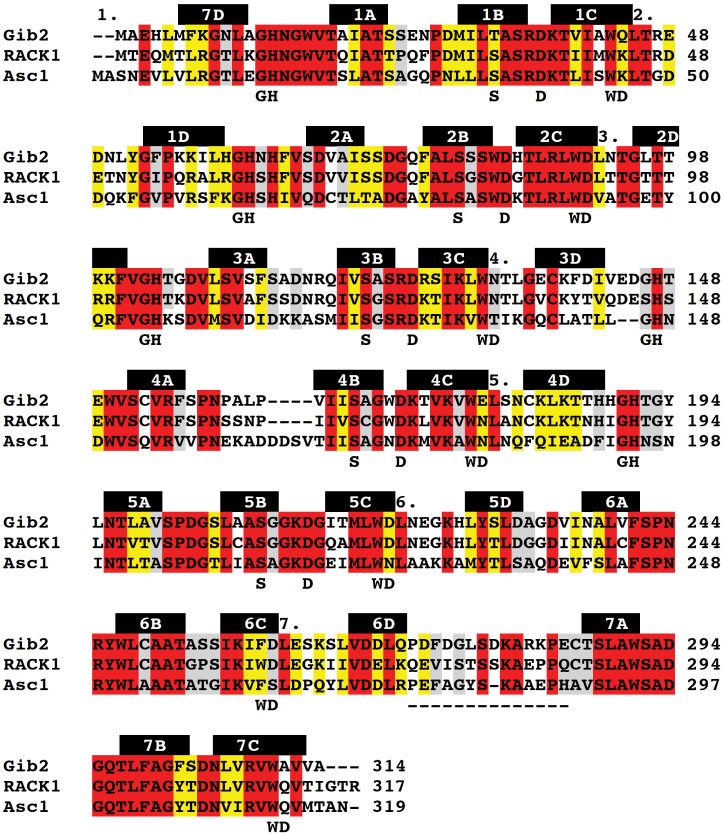
Sequence alignment of *C. neoformans* Gib2 (UniProt accession number A0AUJ0), *H. sapiens* RACK1 (P63244), and *S. cerevisiae* Asc1 (P38011). Multiple sequence alignment was performed using ClustalW2 (v2.1). All fully conserved residues are highlighted in red. Conserved residues with high (scoring > 0.5 in the Gonnet PAM 250 matrix) and moderate (scoring ≤ 0.5) similar properties are highlighted in yellow and grey, respectively. The WD motifs are numbered above the sequences, and the positions of the conserved WD and GH repeats, as well as structurally conserved S and D residues in WD proteins are shown below the sequences. The locations of β-sheets forming the propeller blades in Gib2 are indicated above the sequence as black bars, and the extended loop residues are highlighted in the dashed line below the sequence.

**Figure 2:**
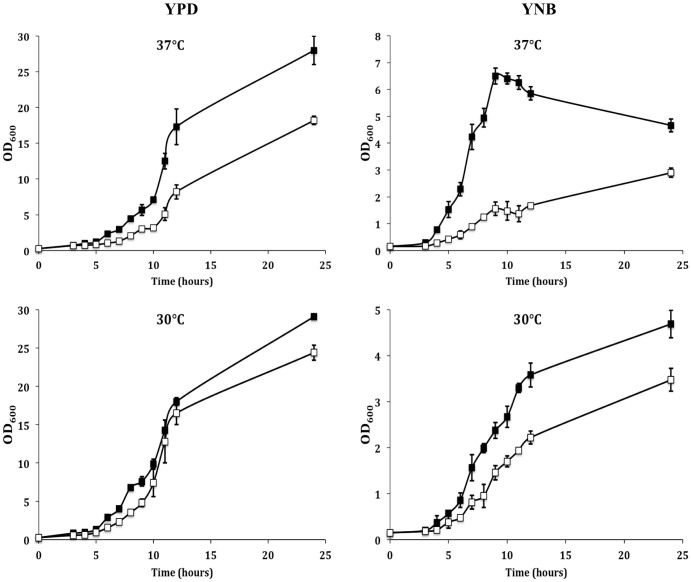
Gib2 is required for the growth of *C. neoformans*. The wild-type H99 (filled squares) and *GIB2* knockout strains (empty squares) were grown in YPD (left panel) and YNB (right panel) media at 37°C (top panel) and 30°C (bottom panel). OD_600_ was monitored to represent growth. The experiments were performed in three biological replicates, and standard deviations are shown.

We determined the crystal structure of Gib2 at 2.2-Å resolution by molecular replacement using Asc1 (PDB ID: 3FRX^29^) as a search model. The asymmetric unit contained one copy of Gib2. Data collection and refinement statistics are given in Table 1. The model (Fig. 4) includes all of the *C. neoformans* Gib2 residues (314 in total), except for Met1. The electron-density map is well defined apart from the first and last residue, as well as a short stretch of side chains between residues Gly276 and Arg282 in the extended loop linking blades six and seven (Fig. 1 and Fig. 4). the structure features the predicted seven β-propeller fold, with an overall shape that resembles a donut (Fig. 4) of approximate dimensions 45 Å and 10 Å of the outer and inner circle, respectively, and 30 Å in width. When observed from the side, one rim is slightly narrower, resulting in a conical overall appearance. Moreover, all of the seven β-propeller blades are arranged radially around the central axis and comprise four twisted antiparallel β-sheets labelled A, B, C, and D (Fig. 1 and Fig. 4), starting from the inside. Neighbouring blades are connected by loops linking D and A sheets, along with loops connecting inter-blade B and C sheets, which are exposed at the narrower rim. The loops connecting inter-blade A and B, as well as C and D sheets, are exposed at the larger rim interface of the propeller (Fig. 1 and Fig. 4).

The interactions stabilising the β-propeller fold are conserved in blades one to five. The aromatic side chains of the conserved Trp residues of the WD repeat (Fig. 1) point to the hydrophobic space between the blades and make interactions with Ser (Thr in blade one) ten residues downstream through hydrogen bond (Fig. 5). The Ser residue also makes hydrogen bond interactions with His residues from the conserved GH motifs in the loops connecting neighbouring blades, which in turn contacts with conserved Asp six residues downstream of the Trp through hydrogen bonds (Fig. 5). The network of inter-blade hydrogen bonds between conserved residues observed in blades one to five is absent in blades six and seven. In blade six, Phe residue replaces the WD motif Trp residue. Although the corresponding Trp residue is present in blade seven (Fig. 1), its orientation differs from the one observed in blades one to five. Deviation from the conserved structural motif in blades six and seven might be necessary to accommodate the extended loop located between these two blades (Fig. 4) or possibly provide a more dynamic binding site for protein partners.

### Gib2 structure comparisons

The structure of *C. neoformans* Gib2 showed good agreement with those of human RACK1 (PDB ID 4AOW)^30^ and *S. cerevisiae* Asc1 (PDB ID 3FRX)^29^ with Gib2 being more similar to RACK1 with an RMSD of 0.43 Å for 1919 superimposed atoms (Fig. 6). The main difference is observed in the outer (D) β-sheets that are shorter in Gib2 (Fig. 6A) compared to Asc1 and RACK1. Additionally, the inner (A) β-sheets are slightly more centrally oriented in Gib2 (Fig. 6B). As the extended loop between blades six and seven displays higher sequence variability among Gib2, RACK1, and Asc1 compared to that in other regions (Fig. 1), it likely exhibits a higher degree of flexibility in protein structures. In accordance, this region has a less well-defined electron density map for side chains of various residues in both Gib2 (Leu277 to Arg282) and Asc1^29^ and was not visible in the electron-density map of RACK1^30^. Nonetheless, the electron density for the backbone of the extended loop was sufficient to shed light on the overall conformation of the loop region in Gib2 (Fig. 6C). Although the middle portion of the loop seems to have a different conformation in Gib2 and Asc1, the knob-like structure in Asc1 consisting of stacked Pro276, Phe278, and Pro287^29^ is also observed in the Gib2 structure (consisting of Pro272, Phe274, and Pro284, respectively) (Fig. 6D). This knob-like structure is most likely not present in RACK1^30^, as the corresponding residues are Gln272, Val274, and Pro284, respectively (Fig. 1). The loop region is likely less flexible in Asc1 because it contains one less residue and is further stabilised by an edge-to-face π-π interaction involving Tyr281^29^. The latter is replaced by Leu and Thr in Gib2 and RACK1 proteins, respectively (Fig. 1).

Although Gib2 crystallises as a monomer, Western blotting has revealed that it can form dimeric complexes under physiological conditions^14^. The structural characterisation of yeast ribosomes showed that Asc1 co-purifies with ribosomes in the monomeric form^31^. However, the crystal structure of the Asc1 homodimer revealed that, at least in solution, oligomerisation could occur through the reorganisation of blades four of both monomers, creating a shared blade and exposing a different surface to potential binding partners^32^. A small fraction of human RACK1 protein is also present in the oligomeric form, mostly as dimers in solution^33^. The proportion of dimeric RACK1 seems to depend on conditions, such as salt concentration, pH, and temperature^33^. Deletion analyses revealed that homodimerisation involves blade four in mammalian RACK1 as well^34^. Whether Gib2 oligomerises in a manner similar to Asc1 and RACK1, as well as the functional significance of oligomerisation, remain to be determined.

### Model for Gib2 bound to the ribosome

Cryo-EM studies of *S. cerevisiae* ribosomes indicted that Asc1 is located in the head region of the 40S subunit close to the mRNA exit tunnel^25^,^29^. Further comparison with other available cryo-EM and crystal structures of eukaryotic ribosomes confirmed that the location and orientation of RACK1 orthologues on ribosomes are conserved in eukaryotes^25^,^31^,^35^,^36^. We superimposed the Gib2 structure onto Asc1 in the presence of the crystal structure of *S. cerevisiae* ribosome (PDB: 3U5B and 3U5C)^31^ and found that the interactions are likely to occur between Gib2 blades one and two, the negatively charged phosphate backbone of ribosomal small subunit RNA helices 39 and 40, and ribosomal proteins rpS16e and rpS17e (Fig. 7). An interaction between Gib2 blade five and the C-terminal tail of rpS3e is also likely, as it can be observed in the crystal structure of *Tetrahymena thermophila* 40S ribosome^35^. It should be stressed, however, that the structural similarity of *C. neoformans* ribosomes to other eukaryotic ribosomes is not known.

### Gib2 interacts with eIF4A

**Figure 3:**
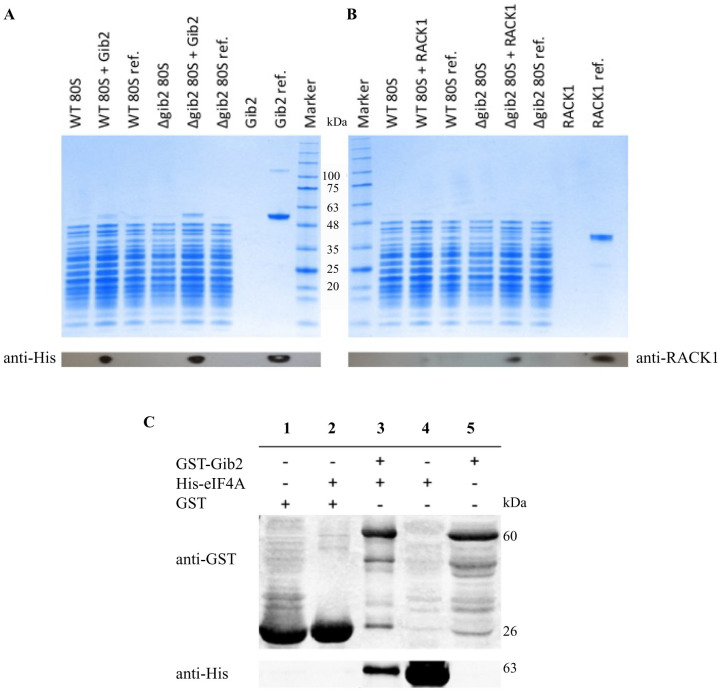
Gib2 interaction with ribosomes and eIF4A *in vitro*. Ribosomes (80S) from the *C. neoformans* wild-type (WT) and *GIB2* knockout (*Δgib2*) strains were incubated with *C. neoformans* Gib2 (A) and human RACK1 (B) proteins and centrifuged through a 1.1-M sucrose cushion. Proteins were then separated using SDS-PAGE. Western blot assays using anti-His and anti-RACK1 antibodies were performed to visualise Gib2 (~50 kDa) and RACK1 (~40 kDa), respectively. For reference (ref.), ribosomes were directly (without sucrose cushion centrifugation) loaded onto the gel. (C) Gib2 interacts with eIF4A *in vitro*. GST-Gib2 and His-eIF4A were expressed in *E. coli* and purified by affinity chromatography. Co-immunoprecipitation was performed as described in the Methods using GST-Gib2 as input. GST protein was used as a negative control. Proteins were separated by SDS-PAGE and analysed by Western blotting using anti-His and anti-GST antibodies to visualise eIF4A and Gib2, respectively. Lanes 4 and 5 are His-eIF4A (~63 kDa) and GST-Gib2 (~60 kDa) protein references, respectively.

The D-E-A-D (Asp-Glu-Ala-Asp)-box containing RNA helicases are essential ribosomal components serving as initiation factors in protein translation in eukaryotic cells^37^. An interaction between Gib2 and the *C. neoformans* eIF4A (eukaryotic translation initiation factor 4A) homologue was established previously by Wang et al^14^. To elaborate the association between Gib2 and the ribosome, we assayed the interaction between Gib2 and eIF4A through the co-immunoprecipitation of heterologously expressed proteins. Consistent with the previous study^14^, eIF4A (expressed in pET-32a, instead of pRSET-B) was pulled down by Gib2 (expressed in pGEX-6P, instead of pET41a(+) (Fig. 3C).

## Discussion

*C. neoformans* is a fungal pathogen that causes life-threatening infections primarily in individuals with compromised immune systems. The virulence of *C. neoformans* is regulated by the cAMP-dependent signalling pathway^4^,^5^,^6^,^7^,^8^. Although Gib2 has been reported to have a role in cAMP signalling by promoting cAMP levels in cells lacking G protein α subunit Gpa1, a key factor in cAMP-dependent regulation of virulence^13^,^14^, disruption of the *GIB2* gene in *C. neoformans* serotype A, affected neither cAMP levels nor pigment and capsule formation^14^. Nonetheless, murine virulence assays revealed that the *GIB2* deletion strain infected mice had a longer survival than those infected by the wild-type strain^14^. This seemingly conflicting observation suggests that Gib2 regulates virulence characteristics indirectly. Our study validates the previous findings by others and further elevates the study by presenting the crystal structure of Gib2, a key regulatory protein in *C. neoformans*.

**Figure 4:**
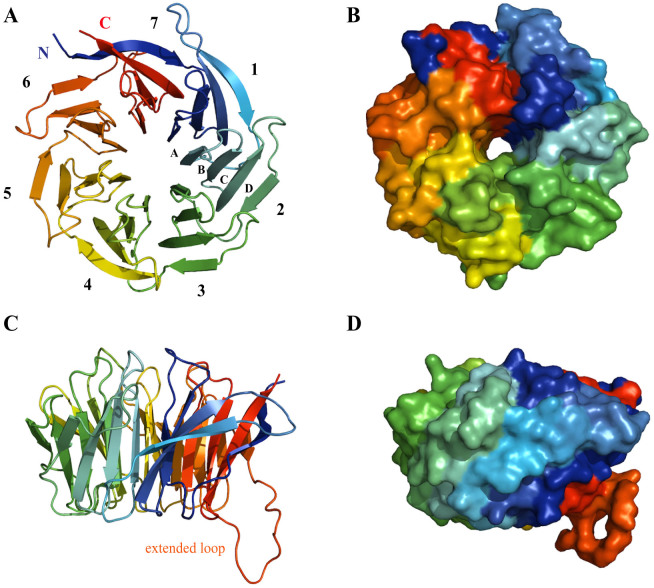
The crystal structure of Gib2. Cartoon (A and C) and surface (B and D) representation of the *C. neoformans* Gib2 crystal structure viewed from the top (A and B) and the side (C and D). Molecules are coloured using the chainbow scheme from blue (N-terminus) to red (C-terminus) and visualised using MacPyMOL software. The seven β-propeller blades are numbered, and individual β-sheets for the second blade are also labelled (A). The extended loop between blades six and seven is also indicated (B).

**Figure 5:**
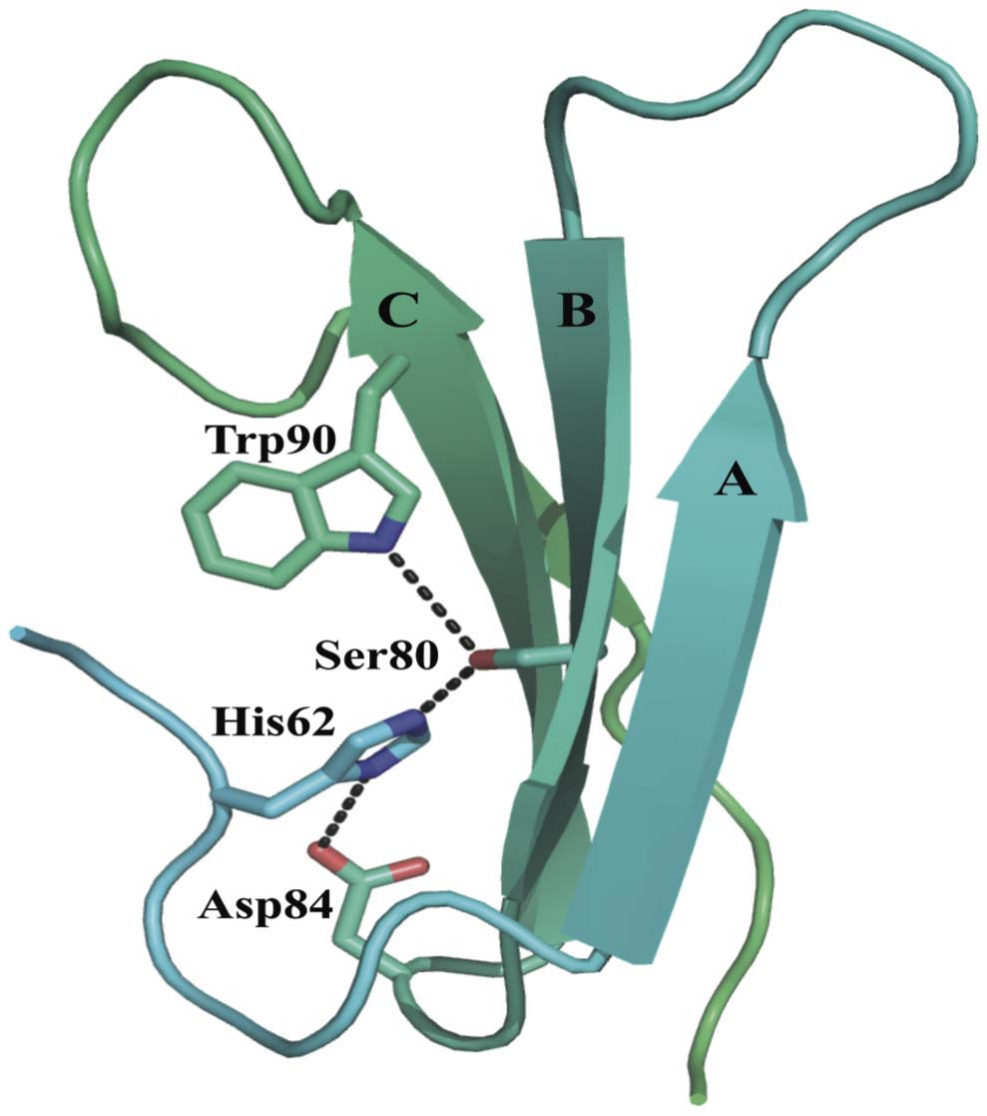
Conserved interactions in Gib2 blades one to five. Blade two is shown with individual β-sheets labelled. The side chains of conserved residues are shown in sticks, and the tertiary interactions stabilising the blade are shown in the dashed line.

**Figure 6:**
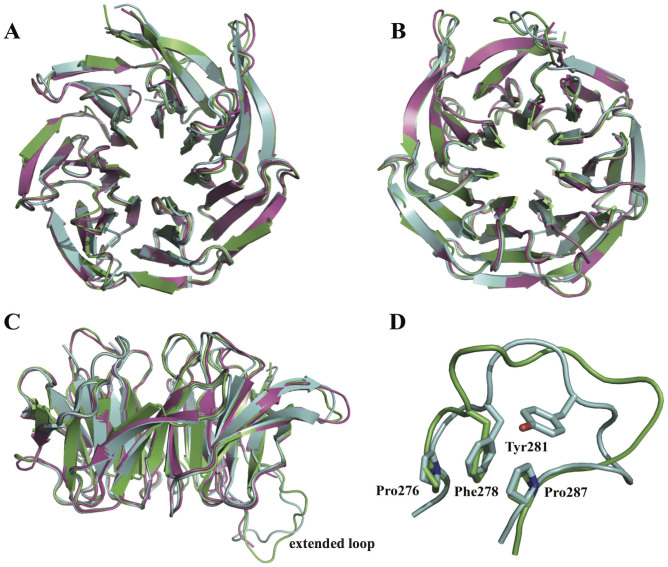
Gib2 comparison with RACK1 and Asc1. Superimposition of *C. neoformans* Gib2 in green, human RACK1 (PDB ID 4AOW) in purple, and *S. cerevisiae* Asc1 (PDB ID 3FRX) in light blue. A, B, and C viewed from the top, bottom, and side, respectively. Close up of the extended loop region (D) is also shown. Side chains of the Asc1 residues involved in the knob-like structure are shown as sticks and labelled individually.

**Figure 7:**
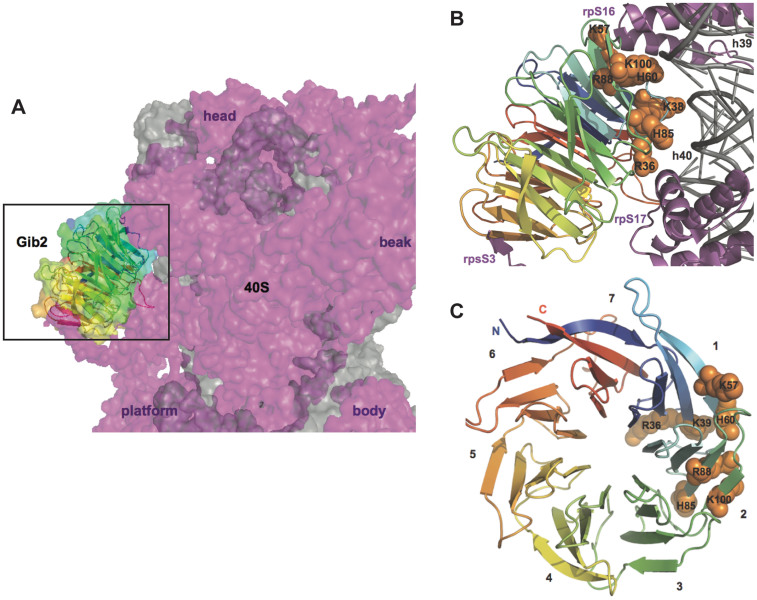
A model of Gib2 interaction with the ribosome. The Gib2 structure was superimposed onto Asc1 in the presence of *S. cerevisiae* ribosome (PDB: 3U5B and 3U5C)^31^ using Coot^56^. (A) The model positions Gib2 to the “back” of small subunit head. The 40S subunit is shown from the side opposite from the mRNA tunnel exit with the 60S interacting interface pointing to the right. Landmarks of the 40S subunit are indicated to aid orientation. Gib2 is shown both as a cartoon and as a surface with same colouring schemes as in Figure 4. The ribosome is shown as a surface rendition with rRNA and r-proteins in grey and purple, respectively. (B) A close-up view of the Gib2 ribosome interface. Conserved and positively charged residues of Gib2 predicted to interact with the ribosome are labelled and shown as orange spheres. The ribosome is shown as a cartoon representation with the same colouring as in panel A, and regions involved in Gib2 binding are labelled. (C) Top view of Gib2 indicating the location of residues predicted to be involved in ribosome binding. β-propeller blades are numbered.

Based on its homology to mammalian RACK1 and yeast Asc1 (Fig. 1), well-known scaffold proteins linking several signalling pathways^17^,^22^, Gib2 has been predicted and shown to have multiple functions in *C. neoformans*^13^,^14^. We propose that Gib2 is structurally similar to RACK1 and Asc1 and that Gib2 is associated with ribosomes as well. Indeed, we were able to determine the crystal structure of *C. neoformans* Gib2 at a 2.2-Å resolution. The Gib2 structure features the β-propeller fold with each of the seven blades consisting of four antiparallel β-sheets (Fig. 4) that show overall good agreement with both Asc1 and RACK1 structures (Fig. 6). In addition, we showed that both the *C. neoformans* Gib2 and the human RACK1 can form a complex with *C. neoformans* ribosomes *in vitro* (Fig. 3). Furthermore, based on the crystal structure of yeast ribosome in complex with Asc1^31^, we modelled the interaction between Gib2 and ribosome (Fig. 7). In *S. cerevisiae* Asc1, there are several conserved, positively charged, and solvent accessible residues that serve as the main association sites for ribosomal binding, e.g., the conserved Arg38-Asp39-Lys40 within the first WD-40 domain are of significance, underlined by the finding that this region is responsible for the decrease in tolerance to translation inhibitors^25^. Intriguingly, these residues are also present in Gib2 (Fig. 7B). Asc1 Arg38 (Arg36 in Gib2) and Lys40 (Lys38) contribute to ribosome binding both in *in vitro*^25^ and in *in vivo* binding assays^29^. Mutations of Lys62, Lys87, Arg90, and Arg102 (correspond to His60, His85, Arg88, and Arg100 in Gib2, respectively) caused defects in Asc1-ribosome association *in vivo*^29^. Lys40, Lys87, Arg90, and Arg102 are believed to form salt bridge interactions with the sugar-phosphate backbone of rRNA, whereas Arg38 interacts with Asp27 in ribosomal protein rps17e^31^,^35^. Although the knob-like structure of the extended loop of Asc1 (Fig. 6) is located close to the ribosome-binding interface, mutations or deletions introduced to this region did not affect ribosome binding *in vivo*^29^. The cryo-EM structure of the canine 80S ribosome also indicated that residues corresponding to Arg36, Lys40, and possibly Arg57 (RACK1 amino acid sequence is identical in mammals) interact with rRNA helices 40 and 39^36^. Although Arg57, Lys62, Lys87, and Arg102 (Asc1 numbering) are not fully conserved, they are mostly replaced by other positively charged residues in RACK1 orthologues (Fig. 1). Based on sequence (Fig. 1) and structural (Fig. 6) similarity to mammalian RACK1 and yeast Asc1 discussed above, we propose that Gib2’s positively charged and surface accessible residues Arg36, Lys38, Lys57, His60, His85, Arg88, and Arg100 contribute to the interactions with rRNA (Fig. 7).

The orientation of previously studied RACK1 orthologues^25^,^29^,^31^,^36^ and, therefore, highly likely Gib2 on the ribosome, suggests that, while binding *per se* could stabilise the 40S subunit, it should not significantly affect ribosome functioning in translation. However, the larger rim face and the sides of blades four to seven of ribosome-bound RACK1 orthologues are solvent accessible and, hence, good candidates for creating binding sites for several protein interaction partners. Moreover, the side of blades five and six of ribosome-bound RACK orthologues face the mRNA exit tunnel. This implicates a potentially significant functional consequence of Gib2-ribosome binding.

There are several examples of protein binding to the four to seven blade region of RACK1/Asc1 proteins. For instance, protein kinase C (PKC) can bind to blade six as revealed by peptide mapping studies^38^,^39^. In addition, a recent affinity grid-based cryo-EM study of PKC binding to RACK1 on the ribosome suggests that it binds to the blade three and four region as well^40^. Although a physical interaction between Pkc1 (PKC homologue in *C. neoformans*) and Gib2 has been reported^13^, the region of Gib2 involved in complex formation needs further identification. Additionally, Src kinase binds to and phosphorylates conserved Tyr residues located at the edge of blades five and six in RACK1^41^,^42^. However, no Src kinase homologues have been identified in *C. neoformans*. Other findings, such as the effect of phosphorylation of RACK1 on its interactions with β-integrins^43^,^44^and Kindlin-3 involving blades five to seven^45^, cannot be validated because of the lack of comparable homologue proteins in *C. neoformans*. However, eukaryotic translation initiation factor 3 (eIF3) was reported to associate with ribosomes through binding to the one to three blade region of Asc1^46^,^47^. It is conceivable that such a binding pattern would also apply to Gib2 because the interaction between Gib2 and eIF4A was validated under two different testing conditions (Fig. 3 and Wang et al^14^). Modelling of the Asc1 homodimer on the 40S subunit also revealed the feasibility of multimeric complex formation^32^, suggesting that Gib2 could employ similar oligomerisation strategies to regulate the recruitment of binding partners to the ribosome.

Examples of the influence of ribosome-bound RACK1 on translation in different eukaryotes are accumulating. For instance, RACK1 stimulates translation by recruiting activated PKC to 40S subunits, and the PKC dependent phosphorylation of eIF6 on 60S subunits leads to subunit association^48^. Accordingly, heterozygous RACK1 gene depletion in mice caused the accumulation of monosomes and impaired protein synthesis^49^. RACK1 also recruits the stress induced c-Jun N-terminal kinase (JNK) to ribosomes, where it phosphorylates the eukaryotic translation elongation factor 1A isoform 2 (eEF1A2) and promotes the degradation of newly synthesised polypeptides (NSP), thereby establishing a role for RACK1 in the quality control of NSPs under stress conditions^50^. In *S. cerevisiae*, the deletion of the *ASC1* gene affected the phosphorylation of eukaryotic translation initiation factors 2 (eIF2) and 4A (eIF4A), affinity of eIF3 and eIF5 binding to 40S ribosomes, and assembly of the pre-initiation complex. These findings indicate an important regulatory role of Asc1 in general translation initiation^46^, and provide compelling reasons for the presence of similar functions by Gib2.

In addition to affecting general translation, RACK1 and Asc1 were shown to regulate the translation of specific mRNAs^51^. For instance, RACK1/Asc1 regulates the translation initiation of specific mRNAs through their respective 5’ UTR sequences^23^, which could be mediated by interactions between RACK1 and ZBP1, or Asc1 and Sc160p^51^,^52^. RACK1/Asc1 could therefore mediate the delivery of specific mRNAs close to the mRNA binding site on 40S ribosome. Based on the high conservation, it is conceivable that Gib2 may also exhibit similar functions.

An interactome analysis showed that Gib2 interacts with more than 50 proteins^14^. In addition to numerous proteins involved in signalling (e.g., Pkc1, Cac1, Ras1), response to chemical stimuli (e.g., Gpa1), transport, and various other cellular processes, a significant proportion of Gib2 binding partners are ribosome related, either ribosomal core components (e.g., RPS3, RPS7, RPL4, RPL6, RPL13, and RPL19) or translation factors (e.g., eEF1A, eIF4)^14^. Therefore, we propose that the structure of Gib2 provides a platform for multiple binding partners and allows the ribosome-bound Gib2 to function as a hub linking diverse cellular processes (e.g., signalling, stress response, intracellular trafficking) to translation in *C. neoformans*.

Thus, Gib2 has both ribosome-independent and -dependent functions in *C. neoformans*. Gib2 interacts with G protein α subunit Gpa1 and assists its functions in the cAMP-signalling pathway to regulate virulence (melanin pigment and capsule formation) of *C. neoformans*^13^,^14^. We suggest that ribosome-bound Gib2 may regulate translation by responding to environmental changes (e.g., higher temperature upon infecting mammals) through interacting with proteins involved in various signalling pathways. Gib2 could affect the functioning of translation factors leading to changes in translation efficiency, the recruitment of specific mRNA to ribosomes, or intracellular trafficking of ribosomes. In the absence of Gib2, *C. neoformans* may face challenges in adjusting to changes in the living environment, leading to reduced fitness and virulence.

Finally, Gib2 is emerging as a link between diverse cellular processes, virulence, and translation regulation in the widely spread and precarious human pathogen *C. neoformans*. Solving the crystal structure of Gib2 sheds light onto its versatile functions as a ribosome-bound scaffold for numerous binding partners. It also provides a basis for future studies, such as the mutagenesis of Gib2, binding assays to identify/confirm its binding partners, the structural characterisation of these protein complexes to reveal the virulence mechanism, and drug target identification for antifungal therapy.

## Methods

### Strains, media, and growth conditions

*C. neoformans* wild-type H99 and *GIB2* deletion strains were grown in 5 L of YPD (2% glucose, 1% yeast extract, 2% bactopeptone) medium at 30°C with shaking until OD_600_ reached 0.6–0.8. Cells were precipitated by centrifugation (3500 rpm for 15 min at 4°C), frozen in liquid nitrogen, and stored at −80°C.

To monitor growth, 100 ml of YPD or YNB (6.7 g of Yeast Nitrogen Base supplemented with 2% dextrose in 1 L of distilled water) medium was inoculated with a single fungal colony and grown overnight at 30°C. Three replicates of 100 ml YPD or YNB media were then inoculated with the overnight culture to OD_600_ 0.15 and grown either at 30°C or 37°C for 24 hours.

The pOPTHis-Lip-Gib2 plasmid was constructed for recombinant Gib2 expression, in which a TEV-cleavable His-tag was placed at the N-terminus. The human RACK1 gene was cloned, and the protein was purified as previously described^30^.

### Ribosome isolation

Three to five grams of fungal cells were re-suspended in 2 ml of buffer A (50 mM Tris-HCl pH 7.0, 50 mM NH_4_Cl, 10 mM magnesium acetate, 100 mM EDTA, 5 mM DTT, 0.2 mM PMSF, and 10% glycerol) and transferred in 1-ml aliquots into 2-ml cryo tubes containing 0.5 mg of glass beads (460 μm). Cells were lysed using a Precellys 24 (Bertin Technologies, France) tissue homogeniser (6400 rpm, three times for 60 sec).

The crude extract was precipitated by centrifugation at 3,000 rpm for 1 min. The lysate was centrifuged at 5,000 rpm for 10 min, and the supernatant was centrifuged twice at 18,000 rpm for 15 min. The supernatant was then centrifuged at 50,000 rpm for 3 hours. The pellet was re-suspended in buffer B (buffer A with 500 mM KCl) and centrifuged at 5,000 rpm for 10 min. The supernatant was overlaid on a 25% glycerol cushion in buffer B and centrifuged at 50,000 rpm for 3 hours. The pellet was re-suspended in buffer A with 10% sucrose and centrifuged at 5,000 rpm for 10 min. The supernatant was diluted two-fold with buffer A (without sucrose or glycerol), layered onto 10 to 30% sucrose gradient in buffer A and centrifuged at 19,000 rpm for 17.5 hours using SW 28 Ti type rotor (Beckman Coulter Inc. US).

The ribosome profile was determined by continuous monitoring of A_260_, and 80S ribosome-containing fractions were pooled. Ribosomes were precipitated by centrifugation at 40,000 rpm for 20 hours, re-suspended in buffer G (10 mM Hepes-KOH pH 7.5, 50 mM KOAc, 10 mM NH_4_Cl, 5 mM Mg(OAc)_2_, and 2 mM DTT), and stored at −80°C.

### Gib2 expression and purification

BL21(DE3)pLysS competent cells were transformed with the pOPTHisLip-Gib2 plasmid and grown in 5 L of 2YT media (16 g of Bacto tryptone, 10 g of Bacto yeast extract, and 5 g of NaCl per 1 L) supplemented with ampicillin and chloramphenicol. When the OD_600_ reached 0.3, the temperature was lowered to 16°C; when the OD_600_ reached 0.8–0.9, isopropyl β-D-1-thiogalactopyranoside (IPTG, 2.5 mM) was added to induce Gib2 expression. Cells grown overnight were harvested by centrifugation and stored at −80°C.

Cells were re-suspended in lysis buffer (50 mM Tris-HCl pH 8.0, 300 mM NaCl, and 5 mM β-mercaptoethanol) and lysed using a Panda homogeniser (GEA Niro Soavi, Italy). The lysate was centrifuged at 20,000 rpm for 20 min. The supernatant was the loaded onto a HisTrap HP 5-ml column (GE Healthcare, UK) equilibrated with the same buffer. Imidazole was used for protein elution. Gib2-containing fractions were pooled and dialysed overnight at 4°C against buffer containing 50 mM Tris-HCl pH 8.0, 50 mM NaCl, and 5 mM β-mercaptoethanol. The TEV protease (Sigma-Aldrich, US) was added to remove the His-tag, and the purification process was repeated using 50 mM NaCl instead of 300 mM. The flow through was collected. Alternatively, dialysed Gib2 fractions were loaded onto a HiTrap Q 5-ml column and an increasing NaCl concentration was used for protein elution. Gib2-containing fractions were pooled and concentrated to 10 ml before loading onto a HiLoad 26/60 Superdex 75 pg column (GE Healthcare). Column pre-equilibration and protein elution were performed using buffer containing 20 mM Tris-HCl pH 8.0, 50 mM NaCl, and 5 mM β-mercaptoethanol. Gib2 appeared as a single peak and the corresponding fractions were pooled, concentrated, and stored at −80°C.

### *In vitro* ribosome binding assay

80S ribosomes (1.5 μM) were incubated with 6 μM Gib2 (with His-tag, 46.2 kDa) or human RACK1 (~40 kDa) in buffer G in a final volume of 25 μl at 30°C for 25 min. The reaction mixture was filtered through a 0.22-μm filter, the volume was adjusted to 50 μl with buffer G, and layered onto 200 μl of 1.1 M sucrose cushion followed by centrifugation at 45,000 rpm for 18 hours at 4°C. For control, Gib2 and RACK1 proteins without 80S ribosomes were layered onto sucrose cushion in parallel. The pellet was washed once and dissolved in buffer G. Samples were analysed by SDS-PAGE and Western blotting analysis following standard protocols. The anti-His and anti-RACK1 antibodies were used for detecting Gib2 and RACK1, respectively.

### Co-immunoprecipitation

Gib2 and eIF4A cDNAs were cloned into vectors pGEX4T-2 and pET32a, respectively, and transformed into Rosetta 2(DE3) cells (Novagen, US). 1 L of LB medium was inoculated with 5 ml of overnight culture and grown at 37°C until OD_600_ reached 0.5. IPTG (0.1 mM final concentration) was added to induce protein expression at 25°C. After 8 hours, cells were harvested by centrifugation and stored at −70°C. To extract proteins, cells were suspended in lysis buffer (20 mM Tris-HCl pH 7.4, 0.15 M NaCl, 0.5 mM EDTA, 1% Triton X-100, 1 mM DTT, and 1 mM PMSF) and lysed with sonication (2 sec pulses with 4 sec pauses for 2 min). Crude extracts were centrifuged (13,000 rpm for 15 min at 4°C) and supernatants were recovered. In case of GST-tagged Gib2, the supernatant was mixed with glutathione-sepharose resin (Amersham Pharmacia, US) for 2 h. The slurry mixture was then centrifuged at 500 rpm for 2 min at 4°C, washed with Tris-NaCl buffer (50 mM Tris-HCl pH 7.4, 100 mM NaCl, 1% Triton X-100, and 1 mM PMSF), and the protein was eluted by adding 15 mM glutathione. The eluted protein was dialyzed overnight against Tris-NaCl buffer using Slide-A-Lyzer cassette (Thermo Fisher Scientific, US), recovered, and verified by SDS-PAGE and Western blotting analysis with the anti-GST antibody (M20007, Abmart, China). Preparation of the His-tagged eIF4A protein was similar to the above except that crude protein extract was mixed with His-Select Nickel affinity gel (Sigma-Aldrich, US), eluted with Tris-NaCl buffer containing 200 mM imidazole, and dialysed as above. Target protein was verified by Western blotting analysis with the anti-His antibody (M20001, Abmart, China).

For binding, 500 μl of GST-Gib2 protein was added to glutathione-sepharose resin in Tris-NaCl buffer. 500 μl of His-eIF4A protein was then added and the mixture was incubated overnight with gentle rotation at 4°C. The resin was precipitated and washed three times with Tris-NaCl buffer. 100 μl of Tris-NaCl buffer and 25 μl of 5× protein sample buffer were then added to the resin before denaturing by boiling for 5 min. 4 μl of the sample was then separated by SDS-PAGE, transferred to the PVDF membrane, and visualized using the anti-GST and anti-His antibodies. For the negative control, the GST protein was used as an input.

### Crystallisation

The TEV protease-cleaved Gib2 (~9.3 mg/ml) was used for crystallisation trials. Initial crystal hits were found using commercial screens: Crystal Screen, Index, and SaltRx (Hampton Research, US); and Wizard (Emerald Biostructures, US) using the Phoenix protein crystallisation robot (Art Robbins Instruments, US) as sitting drops in 96-well plates. Crystals of ~400 × 100 × 50 μm grew at 18°C when 0.2 μl of the well solution (100 mM cacodylate (pH 6.5) and 1 M sodium citrate) was mixed with 0.2 μl protein sample. For cryo protection, 30% PEG3350 was added. Crystals were mounted and flash frozen in liquid nitrogen.

### Data collection, processing, and model building

Diffraction data were collected on the PXI beamline at the Swiss Light Source (SLS) at a 1-Å wavelength using a Pilatus 6 M detector (Dectris, Switzerland) at 100 K. The data were processed using X-ray Detector Software (XDS)^53^. The structure was solved by molecular replacement with Phaser^54^ using the structure of *S. cerevisiae* Asc1 (PDB ID: 3FRX, Ref. 29) as the search model. Subsequently, aromatic model building was carried out by ARP/wARP^55^. The model was further improved by iterations of manual model building with COOT^56^ and refinement with Phenix^57^. Ramachandran plot statistics were as follows: favoured (94.2%), allowed (5.2%), and outliers (0.6%). Crystallographic data and refinement are summarised in Table 1. All of the structural figures were created using MacPyMOL (www.pymol.org).

## Additional Information

**Accession codes:** The coordinates and structure factors for Gib2 have been deposited in the PDB with accession code 4D6V at 2.2-Å resolution.

## Figures and Tables

**Table 1:** Data collection and refinement statistics

**Data collection**	
Space group	*P4_1_*2_1_2
Cell dimensions	
* a*, *b*, *c* (Å)	81.7, 81.7, 136.0
α, β, γ (°)	90.0, 90.0, 90.0
Resolution (Å)	50.0–2.2 (2.35–2.2)*
*R*_sym _(%)	10.2 (92.7)
*I*/σ*I*	13.5 (2.5)
CC1/2 (%)	99.8 (91.8)
Completeness (%)	99.9 (99.9)
Redundancy	7.7 (5.9)

**Refinement**	
Resolution (Å)	50.0–2.2
No. of reflections	187,760
*R*_work_/*R*_free_ (%)	19.5/23.1
*B*-factors	
Protein	52.1
Water	48.1
R.m.s. deviations	
Bond lengths (Å)	0.009
Bond angles (°)	1.3

*Values in parentheses are for the highest-resolution shell.
